# Depression and anxiety in ovarian cancer: a systematic review and meta-analysis of prevalence rates

**DOI:** 10.1136/bmjopen-2015-007618

**Published:** 2015-11-30

**Authors:** Sam Watts, Philip Prescott, Jessica Mason, Natalie McLeod, George Lewith

**Affiliations:** 1 Faculty of Medicine, Department of Primary Care and Population Sciences, University of Southampton, Southampton, UK; 2 Department of Mathematics, University of Southampton, Southampton, UK; 3 Department of Urology, Royal Shrewsbury Hospitals NHS Trust, Shrewsbury, UK; 4 Faculty of Medicine, University of Southampton, Southampton, UK

**Keywords:** MENTAL HEALTH

## Abstract

**Objectives:**

To systematically review the literature pertaining to the prevalence of depression and anxiety in patients with ovarian cancer as a function of treatment stage.

**Design:**

Systematic review and meta-analysis.

**Participants:**

3623 patients with ovarian cancer from primary research investigations.

**Primary outcome measure:**

The prevalence of depression and anxiety in patients with ovarian cancer as a function of treatment stage.

**Results:**

We identified 24 full journal articles that met the inclusion criteria for entry into the meta-analysis resulting in a pooled sample size of 3623 patients. The meta-analysis of prevalence rates identified pretreatment, on-treatment and post-treatment depression prevalences of 25.34% (CI 22.79% to 28.07%), 22.99% (CI 19.85% to 26.46%) and 12.71% (CI 10.14% to 15.79%), respectively. Pretreatment, on-treatment and post-treatment anxiety prevalences were 19.12% (CI 17.11% to 21.30%), 26.23% (CI 22.30% to 30.56%) and 27.09% (CI 23.10% to 31.49%).

**Conclusions:**

Our findings suggest that the prevalence of depression and anxiety in women with ovarian cancer, across the treatment spectrum, is significantly greater than in the healthy female population. With the growing emphasis on improving the management of survivorship and quality of life, we conclude that further research is warranted to ensure psychological distress in ovarian cancer is not underdiagnosed and undertreated.

**Strengths and limitations of this study**

- This is the first systematic review and meta-analysis to specifically assess and define the prevalence of depression and anxiety in ovarian cancer.
- The majority of studies entered into this review were cross-sectional in design, so the data available do not provide an assessment of the overall proportion of women who suffer from some degree of psychological distress during their cancer journey.
- High levels of heterogeneity were identified for both depression and anxiety suggesting considerable differences in the prevalence estimates across the included studies. This implies that caution is needed when interpreting the conclusions of this study.

## Introduction

Ovarian cancer (OvCa) is the most common gynaecological cancer in the UK and the seventh highest cause of cancer mortality.^1^ ^2^ With an ageing population, the number of OvCa cases is increasing year on year.^3^

The non-specific symptoms of OvCa such as bloating, abdominal pain and appetite change can be subtle and often wrongly attributed to non-cancer morbidity such as irritable bowel syndrome. As a result, the diagnosis of OvCa is often delayed.^4^ This has resulted in over two-thirds of OvCa diagnoses being made subsequent to spread beyond the pelvis.^5^ Metastatic OvCa is less susceptible to treatment, meaning overall 5-year survival for patients with OvCa is less than 45%.^1^

In light of such a substantial and increasing disease burden, the management of survivorship for patients with OvCa is of paramount importance. Such issues revolve around the effective maintenance of quality of life (QoL) throughout the cancer journey, from initial diagnosis to post-treatment follow-up. This is best highlighted by the recent National Cancer Survivorship Initiative's (NCSI) mandate for enhancing personalised and patient-centred care in the UK.^6^ This emphasises the need for the enhanced management of the specific psychological morbidities commonly associated with the diagnosis and treatment of cancer. Two of the most common psychological morbidities experienced by patients with cancer are depression and anxiety.^7^ Patients with cancer with depression and anxiety are at a significantly greater risk of higher mortality rates,^8^ increased periods of hospitalisation and poorer treatment outcomes.^9^

The prevalence rates of anxiety and depression across all cancer populations is significantly higher than in the population at large with between 14% and 56% of all patients with cancer experience these conditions (compared with only 4.7% and 2.6% of the UK population, respectively) at some point in their cancer journey.^10^ ^11^ The evaluation and management of psychological distress in OvCa is not well reported.^12^ The limited research available suggests a high prevalence of depression and anxiety in OvCa with upper limits ranging from 82% and 92%, respectively,^13^ ^14^ but there has been no published systematic review and meta-analysis of prevalence rates.

The onset and progression of psychological distress in patients with cancer, including patients with OvCa, is not likely to be an acute threat that quickly passes but a chronic one with peaks and troughs of severity that occur during key stages of the cancer journey. We do not currently understand at what stage of the OvCa journey, from initial diagnosis to post-treatment follow-up, patients find most distressing. If this information was available, it might allow the healthcare team to ‘risk-adapt’ their psychological screening and support to maximise the chances of patients with OvCa with depression and anxiety being identified, diagnosed and managed in a timely fashion.

The aim of the current meta-analysis was to address these issues and provide an initial estimate of the prevalence of clinical depression and anxiety in patients with OvCa during the three key stages of cancer treatment: pretreatment, on-treatment and post-treatment.

## Methods

### Eligibility criteria

Studies that investigated the specific prevalence of depression and anxiety in patients with OvCa in full journal articles were included. Studies published in conference proceedings, qualitative research, commentaries and discussions, letters, books, book chapters or research not published in the English language were excluded.

Eligible studies were restricted to research focusing on individuals with (1) a biopsy-confirmed diagnosis of OvCa or (2) a clearly defined staging of OvCa. If patients with OvCa were included within an investigation that recruited mixed cancer populations, the study was required to have reported data about the patients with OvCa as a distinct subsample. The primary outcome for the current meta-analysis was the percentage prevalence of depression and anxiety. Thus, inclusion into the meta-analysis was restricted to those studies that specifically reported the prevalence of depression and/or anxiety in patients with a confirmed diagnosis of OvCa in percentage format or a format that allowed for the computation of percentage.

To be eligible for inclusion, each study was required to provide a clear definition of the OvCa treatments undertaken by the study participants and when such treatments took place: (1) pretreatment—treatment that was yet to be undertaken, (2) on-treatment—treatment that was being undertaken at the time of the study or (3) post-treatment—treatment that had already been completed. For the latter category, it was a requirement that the authors specified the time lapse since the cessation of treatment.

### Questionnaire analysis

Entry into the meta-analysis was restricted to data that were collected from questionnaires that provided specific, valid and reliable measurements of depression and anxiety. To enable this, a series of questionnaire-specific inclusion criteria were created against which all of the questionnaires utilised in the studies could be assessed; each questionnaire must:

1. Allow for the specific and independent measurement of depression and anxiety.
2. Have available established threshold information for the diagnosis of depression and anxiety.
3. The concurrent validity of each questionnaire must have been assessed in comparison to established ‘gold standard’ instruments (such as the Hamilton Depression and Anxiety Scale) which have themselves been assessed against clinical interviews or a clinician diagnosis with correlation analyses typically greater than 0.65.
4. The internal validity and reliability of each questionnaire must have been assessed and deemed acceptable (test-retest).

Ten questionnaires meeting the criteria were identified a priori which included the Hospital Anxiety and Depression Scale (44), State-Trait Anxiety Scale 45, Centre for Epidemiologic Studies Depression Scale 46, Beck Depression Inventory (47), Self-Rating Anxiety Scale (48), Self-Rating Depression Scale (49), Brief Symptom Inventory (50), Composite International Diagnostic Interview (51), and the Structural Clinical Interview for Diagnostic and Statistical Manual of Mental Disorders, 4th Edition (DSM-IV; SCID; 52).

### Identifying research evidence

Data searches were conducted between October 2013 and April 2014. We searched six electronic databases (OVID MEDLINE, EMBASE, AMED, PsycINFO, CINAHL and Web of Science) for articles that met the previously discussed criteria using prespecified MeSH terms as that included ‘Ovarian Neoplasm (EXP)’ OR ‘Ovarian Cancer’ AND ‘Depression (EXP)’ or ‘Anxiety (EXP)’ or ‘Psychological distress (EXP)’ or ‘Stress (EXP)’ or ‘Distress (EXP)’.

To supplement the electronic searches, we also conducted searches of the reference lists of previous reviews, key papers and other relevant articles identified by the electronic search. We also conducted systematic searches of the content lists of key journals to identify any additional studies missed by the electronic searches.

### Study selection

**Figure 1:**
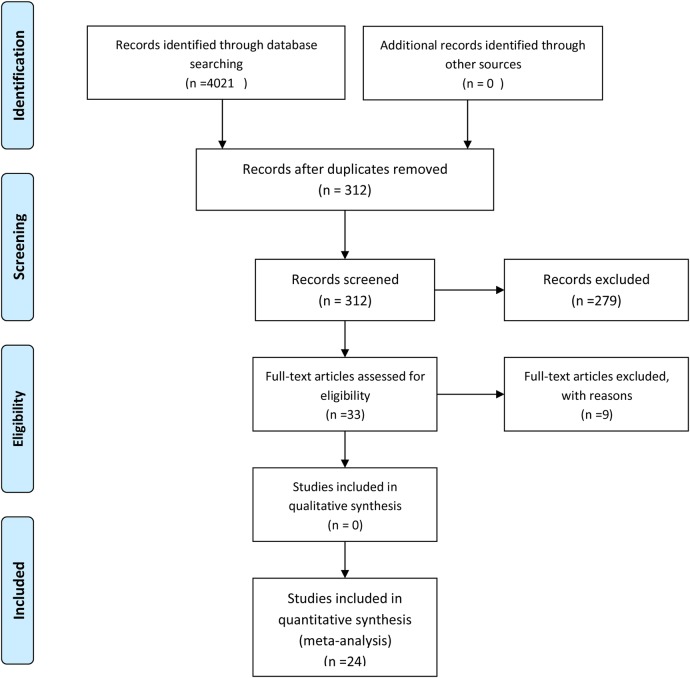
PRISMA 2009 flow diagram.

Titles and abstracts were initially assessed for eligibility. If it was possible to confirm that an article met the inclusion criteria from the abstract alone, the full-text article was retrieved. If it was clear from the abstract that an article was not eligible, it was rejected immediately. If it was not possible to determine the eligibility of an article from the abstract, the full-text article was retrieved. If any key information was missing, we contacted the authors for the missing data. If this was not possible or ineffective, the study was rejected (see figure 1).

### Data extraction

The following specific information relating to data collection and results was extracted individually from each identified article and entered into a predesigned Excel spread sheet: date and geographical location of data collection; aims and objectives of the investigation; study design; participant inclusion and exclusion criteria; recruitment procedures; sample size; disease stage; sociodemographic status (age, ethnicity and relationship, educational and employment status); time since diagnosis; additional comorbidity; stage of treatment (pretreatment, on-treatment or post-treatment); treatments; questionnaires utilised with threshold data; statistical analyses performed; depression prevalence (%) and anxiety prevalence (%).

To test the consistency of data extraction across the studies, three researchers (SW, NM and JM) extracted data from the same six randomly selected articles then compared the results of their extraction. A points system was utilised to allow for the objective assessment of consistency. One point was allocated for variables with identical data extraction and 0 points for variables with differences. Across all ratings, consistency ranged from 91% to 97% (median 94%). Where discrepancy did exist, the entire research team convened to allow for the generation of a final decision as to whether to include a study or not.

### Meta-analysis procedures

The logits of proportions method of conducting the statistical analysis was employed, rather than relying on normal approximations of binomial distributions. A meta-analysis using logits takes into account the sample sizes of the studies to give the overall prevalence estimate as a weighted average with appropriate 95% CIs. If the study, i=1, …, k, has sample size n_i_ and proportion p_i_, the logit of p_i_ is given by logit(p_i_)=log(p_i_/(1−p_i_) with SE 
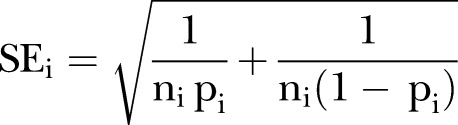
.

The meta-analysis uses weights w_i_=1/SE_i_^2^=n_i_p_i_(1−p_i_) and estimates the logit of the overall prevalence using 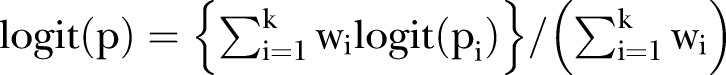. The SE of logit(p) is 
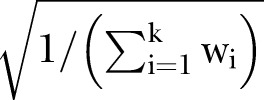
 so that logit(p)±2 
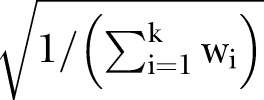
 provides an appropriate 95% CI for logit(p). The meta-analysis estimate and confidence limits are then transformed back using the inverse transformation
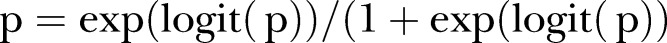
_._

### Test for heterogeneity

Cochran's Q test was applied to the logits to test the hypothesis of homogeneity of the within-study estimates of the proportions, with larger Q values suggesting that the estimates are not homogeneous. Initial analyses highlighted Q values between Q=76.7 and 259.2, with some of the larger values suggesting a considerable degree of heterogeneity. For completeness, meta-analysis results have been provided even for those cases where significant heterogeneity was evident.

## Results

### Search results

The electronic database searches initially yielded 4021 journal article references. In total, 3709 of these were subsequently removed due to either duplication or a failure to meet the inclusion criteria. Full-text articles were then retrieved and critically appraised for the remaining 312 journal references. Of these 312 articles, 279 did not meet the inclusion criteria (224 studies reported generic QoL data rather than providing a specific measure of depression and/or anxiety while the remaining 55 studies provided only data for generic gynaecological cancer rather than OvCa-specific data). Of the remaining 33 acceptable studies, nine authors were contacted to provide additional data. Of these, six authors no longer had the data required and three failed to respond. The remaining 24 articles were entered into the meta-analysis. Hand searches of the key journals identified by the electronic database search and the reference lists of articles identified through the electronic database revealed no further studies (figure 1).

### Study locations

The 24 studies included in the meta-analysis were published between 2003 and 2013. Six of the studies were conducted in Australia,^15–20^ two in Austria,^14^ ^21^ one in the Czech Republic,^13^ two in Norway,^22^ ^23^ three in the UK,^24–26^ one in Japan^27^ and nine in the USA.^28–36^ Eighteen of the studies were cross-sectional in design and seven were longitudinal. An overview of the key features of each of the included studies can be seen in table 1.

**Table 1:** Overview of included studies

Author	Year	Location	Sample size	Participant age	Questionnaire used	Treatment phase
Hodgkinson	2006	Australia	54	58.2	HADS	Post-treatment
Wenzel	2002	USA	49	55.9	CES-D	Post-treatment
Liavaag	2007	Norway	189	59.3	HADS	Post-treatment
Hipkins	2004	UK	63	58.2	HADS	Pretreatment and post-treatment
Bisseling	2009	Australia	62	36.5	HADS	Post-treatment
Norton	2004	USA	143	55.0	BDI	On-treatment
Parker	2006	USA	126	58.7	CES-D	On-treatment
Goncalves	2008	UK	121	61.1	HADS	On-treatment and post-treatment
Costanzo	2005	USA	61	60.1	CES-D	Pretreatment
Price	2010	Australia	798	60.0	HADS	Pretreatment, treatment
Sukegawa	2008	Japan	27	50.1	STAI	On-treatment
Liavaag	2007	Norway	189	57.8	HADS	On-treatment
Price	2009	Australia	613	60.5	HADS	Pretreatment and on-treatment
Goncalves	2010	UK	21	58.8	HADS	Pretreatment and post-treatment
Slovacek	2009	Czech Republic	30	62.1	SDS	On-treatment
Lutgendorf	2009	USA	19	61.0	CES-D and BDI	Pretreatment
Schulman-Green	2008	USA	84	60.8	STAI and CES-D	Pretreatment
Stafford	2010	Australia	71	58.5	HADS	On-treatment and post-treatment
Urbaniec	2011	Australia	21	56.7	BDI and STAI	Post-treatment
Clevenger	2013	USA	301	59.4	CES-D	Pretreatment, on-treatment and post-treatment
Holzner	2003	Austria	98	57.4	HADS	Post-treatment
Lutgendorf	2008	USA	56	63.5	CES-D	Pretreatment
Lutgendorf	2008	USA	86	55.6	CES-D	Pretreatment
Meraner	2012	Austria	55	52.8	HADS	Pretreatment, on-treatment and post-treatment

BDI, Beck Depression Inventory; CES-D, Centre for Epidemiologic Studies Depression Scale; HADS, Hospital Anxiety and Depression Scale; SDS, Self-Rating Depression Scale; STAI, State-Trait Anxiety Scale.

### Study sample sizes

The samples sizes of the studies entered into the review varied widely from 12 to 798. The total sample size across all 24 studies was 3623 with a mean sample size of 151 (table 1).

### Participant age

Data on participant age were reported by all 24 of the studies, and in all cases mean age was reported. The range of mean ages across the 24 studies varied from 19.3 to 88.2 years. The mean age of all participants across the 24 studies weighted by sample size was 58.7 years (2.8; table 1).

### Cancer treatments undertaken

Across the total sample of patients, the treatments undertaken included surgery (n=1852), radiotherapy (n=54), hormone therapy (n=1017), chemotherapy (n=561) and other (n=14). Unfortunately, the basic data collected did not allow us to stratify the treatments undertaken as a function of treatment stage (on-treatment or post-treatment). In the majority of instances, patients who were at different treatment stages were recruited into the same cohort. Thus, these date provide a collective overview of the treatments undertaken by all of the patients, irrespective of treatment stage. Likewise, not all of the included studies provided data on the specific types of treatments undertaken by study participants. Therefore, the sum of each treatment modality is not equal to the overall pooled sample size of this meta-analysis.

### Questionnaires analysis

Of the 10 questionnaires meeting the questionnaire inclusion criteria listed in the Methods section, only 5 were utilised by the 24 studies entered into this meta-analysis. Table 2 lists the five questionnaires, the frequency with which they were used and the clinical cut-off scores utilised to determine caseness.

**Table 2:** Questionnaires utilised in included studies

Questionnaire name	Frequency of use	Clinical cut-off scores utilised
Hospital Anxiety and Depression Scale (HADS)	12	HADS-A: ≥8 HADS-D: ≥8
Beck Depression Inventory (BDI)	3	≥10
Self-Rating Depression Scale (SDS)	1	≥40
Centre for Epidemiologic Studies Depression Scale (CES-D)	8	≥15
State-Trait Anxiety Scale (STAI)	3	≥44

### Meta-analysis of depression and anxiety prevalence

#### Number of studies reporting depression

Of the 24 studies entered into the review, 23 reported data on depression prevalence. Of these, 11 reported depression in pretreatment patients, 10 in on-treatment patients and 10 in post-treatment patients. The total number of studies from the three groups exceeded 23 as several longitudinal studies reported depression in multiple treatment groups (ie, in both pretreatment and on-treatment groups).

#### Number of studies reporting anxiety

Sixteen of the 24 studies entered into the review reported data on anxiety prevalence. Of these 16, 7 reported anxiety in pretreatment patients, 6 in on-treatment patients and 8 in post-treatment patients. The number of total studies from the three groups exceeded 16 as several longitudinal studies reported anxiety in multiple treatment groups (ie, in both pretreatment and on-treatment groups).

#### Number of patients measured for depression

Collectively, measures of depression were recorded from 3464 participants across the 23 studies reporting depression. In terms of the individual treatment groups, 1981 participants provided measures of depression in the pretreatment group, 800 in the on-treatment group and 683 in the post-treatment group.

#### Number of patients measured for anxiety

Collectively, measures of anxiety were recorded from 2636 participants across the 16 studies reporting anxiety. In terms of the individual treatment groups, 1613 participants provided measures of anxiety in the pretreatment group, 481 in the on-treatment group and 542 in the post-treatment group.

### Pretreatment depression and anxiety prevalence

**Figure 2:**
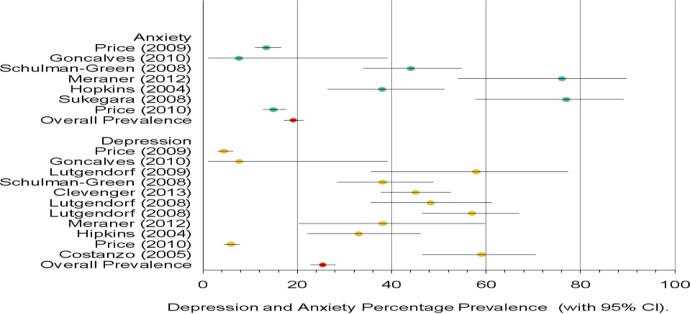
Pretreatment depression and anxiety per cent prevalence.

*Depression*: Within the 10 studies that provided measures of depression in patients with OvCa prior to undergoing treatment (see figure 2), the prevalence of depression was 25.34% (CI 22.79% to 28.07%).

*Anxiety*: Within the six studies that provided measures of anxiety in patients with OvCa prior to undergoing treatment (see figure 2), the prevalence of anxiety was 19.12% (CI 17.11% to 21.30%).

### On-treatment depression and anxiety prevalence

**Figure 3:**
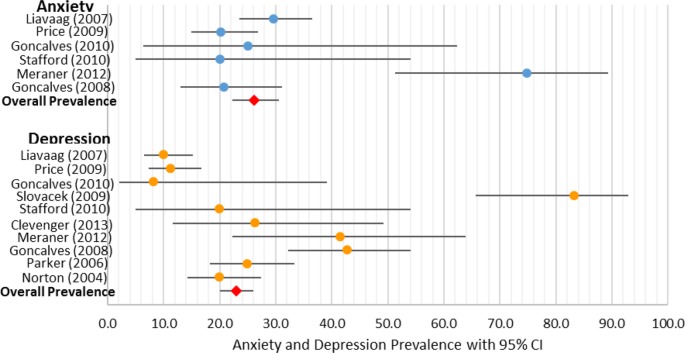
On-treatment anxiety and depression per cent prevalence.

*Depression*: Within the 10 studies that provided measures of depression in patients with OvCa currently undergoing treatment (see figure 3), the prevalence of depression was 22.99% (CI 19.85% to 26.46%).

*Anxiety*: Within the six studies that provided measures of anxiety in patients with OvCa currently undergoing treatment (see figure 3), the prevalence of anxiety was 26.23% (CI 22.30% to 30.56%).

### Post-treatment depression and anxiety prevalence

**Figure 4:**
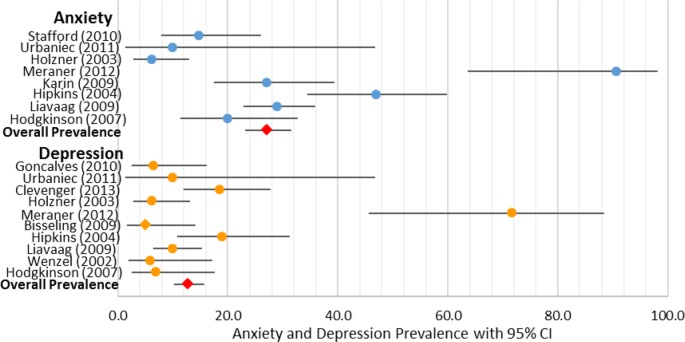
Post-treatment anxiety and depression per cent prevalence.

*Depression*: Within the 10 studies that provided measures of depression in patients with OvCa who had completed treatment (see figure 4), the prevalence of depression was 12.71% (CI 10.14% to 15.79%).

*Anxiety*: Within the eight studies that provided measures of anxiety in patients with OvCa who had completed treatment (see figure 4), the prevalence of anxiety was 27.09% (CI 23.10% to 31.49%).

## Discussion

The diagnosis and management of OvCa is frequently emotionally distressing especially as the prognosis for this cancer is not usually optimistic; this will inevitably impact on the psychological status of these patients.^37^ The results of the current meta-analysis address this issue by providing a systematic overview of the prevalence of depression and anxiety in the patients with OvCa. We have identified a population of 3623 women in largely cross-sectional surveys who have recorded their psychological state utilising valid standardised measures of both anxiety and depression. Our findings suggest that over the trajectory of the OvCa treatment journey, depression was highest before the initiation of treatment (25.3%) before dropping during treatment (23%) and decreasing again after the cessation of treatment (12.7%). In contrast, anxiety was lowest prior to treatment (19.1%) before rising sharply and then plateauing during and after the cessation of treatment (26.2% and 27.1%, respectively). The lifetime prevalence of clinical depression and anxiety in women in the UK is estimated as 10% and 8.2%, respectively.^38^ ^39^ Based on the findings of the current meta-analysis, patients with OvCa in the UK are almost twice as likely to experience clinically significant depression and more than four times as likely to experience clinically significant anxiety as women without OvCa.

Our findings are consistent with the literature reporting depression and anxiety in mixed cancer populations which suggest that between 20% and 30% of patients with gynaecological cancer experience psychological distress at some point during their cancer journey.^40^ Coupled with the large sample size of the current meta-analysis (n=3623), this suggests that our conclusions are consistent with the current literature and offer a robust summary of the data.

There are several limitations to the results generated by this review that need to be taken into account when interpreting the clinical relevance of the findings. It is likely that the onset of psychological distress in women diagnosed with OvCa is not an acute threat but a chronic one with peaks and troughs of severity that vary according to a variety of clinical factors. These may include fear of imminent treatment, treatment-related side effects, fear of progression, actual progression and final transfer to palliative care pathways. The majority of studies in the review were cross-sectional and not longitudinal in design (18 out of 24), so the data available do not provide an assessment of the overall proportion of women who suffer from some degree of psychological distress during their cancer journey. Consequently, the total number of women affected may be higher than we were able to identify from this analysis as people slip in and out of distressed states. We would need to conduct a sustained longitudinal cohort study to address this question. Similarly, there are no consistent mechanisms or gold standard for assessing quality in the predominately cross-sectional studies making up this review that is equivalent to the Cochrane grading system that is largely used for clinical trials. As a consequence, it is very difficult to have any consistent and consensus-based quality assessments in these papers. While the application of our study inclusion criteria helped to provide an objective assessment of study quality, this still represents an important limitation to this study. None of the included studies provided data relating to the patients’ history of depression and anxiety, so it is impossible to determine whether a history of depression and anxiety acted as a significant predictor of current depression and anxiety. Our decision to use only one reviewer for the assessment of titles and abstract during the data identification process also needs to be acknowledged as doing so may increase the possibility of rejecting papers that were suitable for inclusion. High levels of heterogeneity were identified in the meta-analyses for both depression and anxiety suggesting considerable differences in the prevalence estimates across the included studies. Given the variability observed with respect to study sample sizes, the clinical characteristics of the sample, and the different instruments used for assessing depression and anxiety, this heterogeneity was to be expected. Furthermore, this review included two large studies that only provided data for pretreatment and on-treatment patients with OvCa. These studies were very influential on the prevalence estimates generated for these treatment stages.^17^ ^18^ However, these two studies were not included in the post-treatment data set. Thus, when comparing prevalence rates between pretreatment, on-treatment and post-treatment, one must be aware that the same studies were not included in all three treatment stages, making direct comparison difficult. This meta-analysis is based on descriptive uncontrolled data which do not allow us to determine specific causality between OvCa and the reported prevalence of depression and anxiety. Lastly, this study failed to account for or assess publication bias. Our decision to focus our searches wholly on full length journal articles means we may have overlooked relevant and important unpublished and null findings. Standard approaches for addressing publication bias would have been to contact lead authors and researchers in this field to enquire into such relevant unpublished work. However, we had neither the personnel nor financial resources to do so. An interesting finding of the current review was the contrasting patterns of depression and anxiety prevalence rates across the treatment spectrum. While the prevalence of depression decreased significantly from pretreatment to post-treatment follow-up (25.3% and 12.7%, respectively), the opposite was observed for anxiety which increased from a pretreatment prevalence of 19.1% to a post-treatment prevalence of 28%. A possible explanation for this may be that the poor prognostic information that patients with OvCa invariably receive on diagnosis leads to understandable preoccupations with mortality, leading to hopelessness, despair and depression.^41^ However, as patients begin to adjust and come to terms with their diagnosis, these fears begin to dissipate and are replaced with a desire to live life as fully as possible, resulting in a reduction in depression as patients move across the treatment continuum.^41^

However, it is also plausible that those with the highest prevalences of depression in the pretreatment phase were the patients with the most advanced disease with the poorest prognoses. As these patients progressed across the treatment spectrum to post-treatment follow-up, many may have died or become non-responders due to worsening physical health. Therefore, the reduction in depression from pretreatment to post-treatment could have been precipitated by these factors rather to those previously discussed.

In contrast, we observed a sequential rise in anxiety from pretreatment to post-treatment. Lockwood-Rayermann^42^ observed that anxiety in OvCa was significantly correlated to the number of physical symptoms experienced by the patient. Given that the radical treatment options for OvCa all carry substantial morbidity, the significant increase in anxiety from pretreatment to on-treatment may have been precipitated by the treatment-induced symptoms the patients were experiencing.

Anxiety does not diminish after the cessation of treatment but seems to increase slightly. Patients with OvCa experience a marked reduction in clinical consultations after the completion of their treatment as they move into the survivorship phase of the cancer journey. This can lead to feelings of isolation and a fear that their cancer may return or is progressing unobserved. This is a particular concern of patients with OvCa given the general lack of outward physical symptoms associated with this cancer and the fact that accurate self-monitoring is extremely difficult. These observations should be more thoughtfully explored with a longitudinal qualitative study that would allow us to better understand and therefore manage depression and anxiety in OvCa across the treatment spectrum.

Likewise, the age range reported in the included studies varied widely from 19.3 to 88.2 years (mean 56.8). It is likely that this extreme variation will have had a substantial impact on the prevalence of depression and anxiety observed. For example, how does the psychological distress experienced by a 19-year-old woman recently diagnosed with OvCa compare to that observed in an 88-year-old? It would be hugely beneficial for future research to address this issue to allow us to understand the important role that age plays on the prevalence of depression and anxiety in this patient population.

Finally and perhaps most importantly, the current review was unable to stratify depression and anxiety prevalence as a function of disease stage, primarily because patients with varying stages of disease were recruited into the same cohort without stratifying the results as a function of disease stage. However, it is highly likely that the prevalence of psychological distress in OvCa is strongly linked to disease stage, and one would hypothesise that those with recurrent metastatic cancer would experience a higher level of distress than those with localised disease. To address this, it is important that future research aims to recruit and stratify patients with varying stages of disease to identify how this issue impacts on the prevalence of depression and anxiety observed.

The systematic data we report, albeit descriptive in nature, suggest that anxiety and depression are significant issues for patients with OvCa. Further investigation is needed to understand the OvCa journey in more detail both qualitatively and quantitatively, so that the issue of psychological distress can be understood and addressed in a more focused and appropriate manner. Clinically depressed and anxious patients with cancer have lower treatment compliance, poorer treatment outcomes, lower QoL, experience increased periods of hospitalisation and have poorer 5-year survival rates than their non-depressed and anxious counterparts.^8^ ^9^ ^43^ Consequently, the timely diagnosis and management of anxiety and depression should be viewed as an important clinical focus for those working with OvCa as a means of enhancing both clinical outcomes and patient QoL.
